# Item Anomaly Detection Based on Dynamic Partition for Time Series in Recommender Systems

**DOI:** 10.1371/journal.pone.0135155

**Published:** 2015-08-12

**Authors:** Min Gao, Renli Tian, Junhao Wen, Qingyu Xiong, Bin Ling, Linda Yang

**Affiliations:** 1 Key Laboratory of Dependable Service Computing in Cyber Physical Society, Ministry of Education, Chongqing, 400044, China; 2 School of Software Engineering, Chongqing University, Chongqing, 400044, China; 3 School of Engineering, University of Portsmouth, Portsmouth, PO1 3AH, United Kingdom; Universitat Rovira i Virgili, SPAIN

## Abstract

In recent years, recommender systems have become an effective method to process information overload. However, recommendation technology still suffers from many problems. One of the problems is shilling attacks-attackers inject spam user profiles to disturb the list of recommendation items. There are two characteristics of all types of shilling attacks: 1) Item abnormality: The rating of target items is always maximum or minimum; and 2) Attack promptness: It takes only a very short period time to inject attack profiles. Some papers have proposed item anomaly detection methods based on these two characteristics, but their detection rate, false alarm rate, and universality need to be further improved. To solve these problems, this paper proposes an item anomaly detection method based on dynamic partitioning for time series. This method first dynamically partitions item-rating time series based on important points. Then, we use chi square distribution (χ^2^) to detect abnormal intervals. The experimental results on MovieLens 100K and 1M indicate that this approach has a high detection rate and a low false alarm rate and is stable toward different attack models and filler sizes.

## Introduction

Recommendation systems are effective and widely used to solve information overload [1]. Although personalized recommendation technology has achieved huge progress in the cold start problem, forecasting precision, diversity-accuracy dilemma, user experience and contextual-based recommendations [2–6], it still suffers from many problems. Shilling attack, in which attackers inject spam user profiles (user profile indicates the user’s rating set of all items) to change the recommendation results, is one of the most serious problems [7–8]. For a collaborative filtering-based recommendation system without defense, the target item is able to top the recommendation list with spam users’ efforts representing only one percent of the list [9]. The injection of spam users’ ratings in e-commerce systems seriously disturbs the system recommendation ranking, and then misguides users from obtaining what they really want. Consequently, the injection will result in a decline of user satisfaction. Shilling attacks are divided into two categories: push attacks and nuke attacks. Push attacks make the target items easier to be recommended. Nuke attacks make the target items harder to be recommended.

Traditional detection methods of shilling attacks are based mainly on the features of user profiles, such as RDMA and Degsim features [10]. From the machine learning perspective, there are supervised and unsupervised detection algorithms [9–16]. These methods are primarily focused on detecting spam users, which has a good result on some specific attack models, but generality is not strong. Zhang et al. [17] and Gao et al. [18] proposed that the ultimate goal of a shilling attack is to make a change in target items. Therefore, they proposed detection methods for abnormal items, and hoped to solve the problem of shilling attack from the item’s perspective. Focusing on attack promptness, Zhang et al. [17] proposed an item anomaly detection approach based on sample average and sample entropy in a time series., Gao et al. [18] divided all items into different types according the features of items' lifecycle and rating numbers. Then, they used a fixed window to divide the time intervals and χ^2^ was utilized to detect abnormal intervals. In their approach, the time interval is divided by a fixed time window; therefore, the different time window sizes will directly influence the effectiveness of the detection. Additionally, the item’s own characteristic varies with time. The detection difficulty increases as the adjacent window’s rating distribution becomes closer, which results from the time window becoming larger. However, the false alarm rate increases as the adjacent windows rating distribution differences become greater, which results from the time window becomes smaller. The detection rate and false alarm rate of this method needs to be further improved. To solve these problems, this paper proposes a dynamic partitioning method for item-rating time series based on important points followed by identifying item abnormal time intervals based on χ^2^.

The rest of the paper is organized as follows. Section 2 describes commonly used recommendation algorithm, shilling attack models and detection algorithms. Section 3 introduces a dynamic partition for item-rating time series method based on important points and its corresponding detection method. Next, the experimental section describes how to select a suitable parameter *k* value for the dynamic partition method and experimental results of abnormal items detection under different attack models, attack sizes, and filler sizes using two MovieLens datasets. Finally, the whole paper is summarized in Section 5.

## Related Work

### 2.1 Shilling attacks

Recommender systems are tools to help users find a portion of useful information from the Internet [19]. However many recommendation approaches are affected by shilling attacks. To solve this problem, some researchers have conducted research on shilling attack models. In their studies, they proposed a basic framework for attacks based on attack purpose, attack size, and preliminary knowledge.

**Fig 1:**
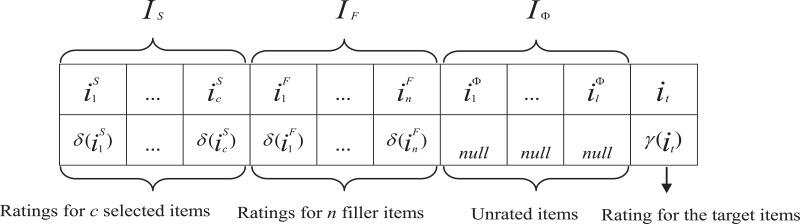
General form of a shilling attack.

Fig 1 shows the general form of a shilling attack [20] where *I*_*S*_ is a set of selected items based on specific needs of the spam user, *I*_*F*_ is a set of randomly selected filler items which the spam user used to disguise himself, *I*_*Φ*_ is a set of unrated items, and *i*_*t*_ is the attack target item.

There are four types of typical attack models.

Random attack model: Selected items are null and the filler items are selected randomly. The ratings of filler items are decided by the overall distribution of ratings in the database.
Average attack model: The attack is the same as with the random attack. Selected items are null and filler items are selected randomly. However, the rating of each filler item is decided by the normal distribution of each item’s own rating average. This attack is effective on user-based collaborative filtering, but not on item-based collaborative filtering.
  Burke et al. [21–22] extended the two types of basic attack models (random and average attack model), additionally proposing the bandwagon attack and the segment attack.
Bandwagon attack model: The selected items are those frequently rated with high ratings. Additionally, spam users also give high ratings to these items to make their own profile look similar to ordinary users. The ratings of the filler items are decided by the overall distribution of ratings in the database. Due to the use of popular items, these profiles are similar to a large number of users. However, this attack model is not very effective on item-based collaborative filtering systems.
Segment attack model: This attack model was proposed for attacking item-based collaborative filtering. The selected items of a segment attack are carefully selected by spam users, being rated with the maximum value, whereas filler items will be rated with the minimum value.

To achieve the attack objective, spam users inject a certain number of user profiles. The strength of the attack event is generally measured by the attack size and filler size. Attack size stands for the percentage of the number of attack user profiles in a recommender system. Filler size stands for the ratio of the numbers of items in a spam user profile to the total items in the recommender system, which describes the item-ratings sparsity degree.

The features of the models are listed in Table 1 [12], where *r*_*max*_ is the maximum value, and *r*_*min*_ is the minimum value in the recommender system.

**Table 1:** Summary of features for four typical shilling attack models.

Attack Type	Attack Model	*I*_*S*_	*I*_*F*_	*I*_*Φ*_	*i*_*t*_
**Random**	Push/nuke	Not used	Ratings assigned with normal distribution around system mean	Determined by filler size	*r*_*max*_ */r*_*min*_
**Average**	Push/nuke	Not used	Ratings assigned with normal distribution around item mean	Determined by filler size	*r*_*max*_ */r*_*min*_
**Bandwagon**	Push	Widely popular items assigned rating *r*_*max*_	Ratings assigned with normal distribution around system mean	Determined by filler size	*r*_*max*_
**Segment**	Push	Items chosen to define the segment assigned rating *r*_*max*_	Ratings assigned with *r*_*min*_	Determined by filler size	*r*_*max*_

### 2.2 Shilling attacks detection methods

Many researchers proposed detection methods for the attack model on collaborative filtering algorithms from the users’ perspective. They promoted the development of anti-shilling attack collaborative filtering technology. For example, Chirita et al. [10] proposed a shilling attack detection method in which five attributes were utilized to recognize attack profiles, including NPD (Number of Prediction-Differences), RDMA (Rating deviation from mean agreement), DegSim (Degree of similarity with top neighbors), Standard Deviation in User’s Ratings and Degree of Agreement with Other User. Burke et al. [11] improved the RDMA to WDMA (Weighted Deviation from Mean Agreement) and WDA (Weighted Degree of Agreement). Additionally, they proposed model-specific attributes to detect the attack profiles. This method was effective for not only random attack detection and segment attack detection but also the detection of small-scale attacks. Burke et al. [22] proposed a series of attributes for the intended model and LengthVar (Length Variance) to improve the entire detection system. Burke et al. [11] regarded shilling detection problem as a supervised classification issue and used generic attributes to detect attacks. Different from the supervised detection method, the unsupervised clustering method was used to discriminate genuine profiles and spam user profiles [1], such as the PCA (Principal Component Analysis) and PLSA (Probabilistic Latent Semantic Analysis) clustering techniques. However, these approaches are limited in obfuscated shilling attacks detection [13]. Wu et al. [23] proposed a Semi-Supervised detector for hybrid shilling attack detection. The method was robust against a hybrid shilling attack, and it improved the accuracy of a recommender system based on collaborative-filtering. These detection methods were used to detect spam users. The detection of spam users is based on a comparison between user profiles. It compares the departure degree of a user profile with other user profiles to determine whether the user profile is a spam user profile. Under different attack models, both the selected user profile features and the deviation between profile features change to obtain good results. However, these detection methods have difficulty achieving good detection results for all of the classical attack models.

With approaches different from those in previous research, Zhang et al. [17] and Gao et al. [18] solved the problem from the items’ perspective. They proposed approaches to detect abnormal item based on the features of item profiles. These methods provided a good approach to solving the shilling attack problem. The method [17] needs to set an initial window size and a parameter *w* (the ratio of fake ratings to the total ratings of target item during an attack event) and used iterative procedures to find an optimal window. However, for a real recommendation system, the parameter *w* is unknowable. An assumption for *w* affects the detection rate. The detection rate of the approach [18] largely depends on the value of a time window. First, the items are divided into four types:Fad items, fashion items, style items and scallop items. Fad items are popular emerging products, but the rating number increases and declines swiftly. Fashion items satisfy few customers at first. However, after some period of time, the rating number grows and declines slowly because these items are gradually accepted by more customers. Style items are basic and typical products that exist for a very long time. Scallop items’ customers increase constantly over time, and most users will give them higher ratings. Then, Gao compared a certain time interval-rating distribution with the rest of the ration distribution of the whole item. The size of the time window is set according to the features of types. However, the item feature changed with time; when the items are initially launched, the feature may behave like a fad type. After some period of time, they may be converted to a style type. In this case, it will take a great deal of time to periodically divide the item types and calculate the time window size.

This paper also focuses on solving shilling attacks from the items’ perspective. To solve those problems, this paper proposes a dynamic partitioning for time series method based on important points followed by applying χ^2^ to find abnormal time intervals.

## Abnormal Item Detection Approach Based on Time Series Dynamic Partitioning

This section describes the steps of the item anomaly detecting approach. Piecewise Linear Representation (PLR) is presented in Section 3.1. We propose an approach based on time series because 1) the purpose of shilling attack is make the target item easier (push attack) or harder (nuke attack) to be recommended. Spam users inject spam user profiles into system to achieve this purpose. 2) Due to the restriction of attack costs, the spam user profiles are injected in a short time and not continued throughout the life cycle of the item. When the item is attacked the number of ratings significant increases. Thus, we can detect target items by using the rating distribution, if the rating numbers of an item significant increase in a short time, we think this item may have been attacked. In order to identify the target item we need find out the intervals which contain spam ratings. We calculate the χ^2^ value to measure the similarity of different time intervals, the intervals containing attacks with others have a low similarity. A good time interval division method maximizes the difference of abnormal intervals with normal intervals. Moreover, although there is some research using a fixed window size to divide the time series, it is difficult to choose an optimal window size and the detection result is influenced by the selected window size. In addition for different item types, the optimal window size is different. Thus, we propose a dynamic item-rating time series partition method based on important points in Section 3.2. This method divides item time series according its own features. Finally, we propose an item anomaly detection method based on dynamic partition for time series.

**Fig 2:**
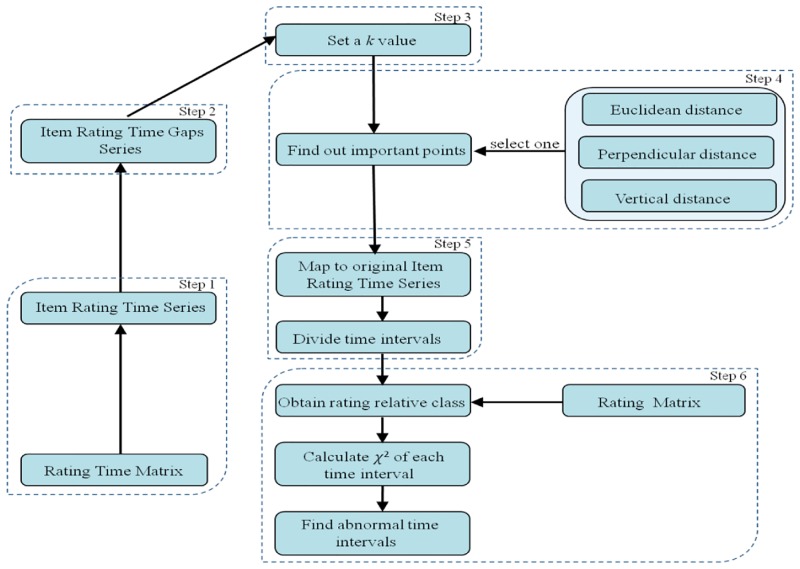
The steps of the item anomaly detecting approach.

The steps of the item anomaly detecting approach are shown as Fig 2.

Step 1: Obtain the item time series according to the rating time matrix.
Step 2: Utilize item rating time series to calculate item-rating time gaps.
Step 3: Set the value *k* (0<*k*≤1), which will decide the termination condition of the searching process for important points.
Step 4: Use the selected k and one distance measurement to find the important points.
Step 5: Mapping the important points to the original item rating time series to divide the item time intervals.
Step 6: Use the rating matrix to obtain a rating relative class for each interval and calculate χ^2^ value of each time interval. Judge whether the χ^2^ value exceeds the threshold. If it does, mark the time intervals as abnormal intervals.

### 3.1 Method of time series piecewise linear representation

To describe the item-rating time series, this paper adopts PLR to represent the time series approximately. PLR is able to compress and filter data [24]. The main problem of the piecewise linear algorithm is to select the appropriate number of line segments and to choose appropriate piecewise points.

To describe the method more clearly in this section, this paper makes several definitions.

#### Definition 1

IRTS (Item-ratings Time Series) is an element-ordered set that is composed of the rating value and the rating time. *IRTS* = {*x*_*1*_ = (*v*_*1*_, *t*_*1*_), *x*_*2*_ = (*v*_*2*_, *t*_*2*_), …, *x*_*n*_ = (*v*_*n*_, *t*_*n*_)}, simply denoted as *IRTS* = {*x*_*1*_, *x*_*2*_, …, *x*_*n*_}. Elements *x*_*i*_ = (*v*_*i*_, *t*_*i*_) indicates that when the time equals *t*_*i*_ in the time series, the rating value is equal to *v*_*i*_, and rating time *t*_*i*_ is strictly increasing (if *i<j*, then *t*_*i*_ *<t*_*j*_).

IRTS has two features: 1) the elements in the time series are small, generally from dozens to hundreds, and 2) the time intervals are not equal, and may differ greatly.

In IRTS, the ratings are generally discrete values. For example, the ratings in MovieLens are the integers from 1 to 5. Thus, if we use rating values to divide the intervals, it irregularity divides the points. Therefore, this paper utilizes item-rating time gaps instead of rating values.

#### Definition 2

IRTGS (Item-ratings Time Gaps Series), *IRTGS* = {(*Gap*_*1*_, *MidT*_*1*_), …, (*Gap*_*n*_, *MidT*_*n*_)}. The computational formulas for *Gap*_*x*_ and *MidT*_*x*_ are as follows,

$$Gap_{x}=t_{x+1}-t_{x}$$ (1)

$$MidT_{x}=\frac{t_{x}+t_{x+1}}{2}$$ (2)

where *t*_*x*_ refers to the rating time. Additionally, because of the huge difference between *Gap* and *MidT*, the IRTGS of all items are normalized by Minimum-maximum standardized (3),

$$x_{i}=\frac{x_{i}-x_{\text{min}}}{x_{\text{max}}-x_{\text{min}}}$$ (3)

where *x*_*i*_ refers to the value of the *i*-th element and *x*_*min*_ and *x*_*max*_ refer to the minimum and maximum values of all elements, respectively.

#### Definition 3

ADNI, the average distance from non-important points to the two important points of a time interval. The formula for ADNI is as (4),

$$ADNI=\frac{\sum_{i=1}^{N}S}{N}$$ (4)

where *N* refers to the total non-important points in a time interval, *S* refers to the distance from *i*-th non-important point in the time interval to the line of the two important points.

### 3.2 Dynamic item-rating time series partition method based on important points

In this method, heuristic rules are utilized to choose the significant time points from time series to divide the time series into subsequences. The method does not restrict the segment number and threshold, but it requires choosing appropriate piecewise points. The steps of the dynamic partition method for item-rating time series are as follows:

Step 1: Given a sequence *P*, extract the first and the last data point *p*_*1*_ and *p*_*n*_ as the two initial important points.
Step 2: If there are points higher than the line from the first important point to the second one, select the highest point from the line between the first important point and the second point as the third important point. If there is no such point, select the point nearest to the line as the third important point.
Step 3: Find the important points between the first important point to the third important point and the third important point to the second important point.
Step 4: Repeat step 3, until two conditions are met. First, there is no data point higher than the line that contains two adjacent important points. Second, the Gap of data points between the two important points are less than *k* (0 <*k*< = 1) times the sum of two important points’.

The purpose of finding important points in this paper is to make rating points which are close enough in terms of rating time series to be classified in the same divided interval.

The first condition in Step 4 is that all non-important points lie under the lines formed by Gap values of two adjacent important points. The reason is that if this rule is broken when there exists non-important points higher than the line, the Gap values of these points are larger than one of the important points. Thus, if we use these points as important points, the points in each time intervals rating feature (close or sparse) will be more similar than the current division. Therefore, if we cannot meet this condition the iteration of the important points search process will not stop.

The second conditions in step 4 is that the Gap values of all non-important points in the same interval are less than *k* times the sum of two important points’. Stopping the algorithm only according to the first condition may lead to early stop iterations while the actual item rating time series changes in different situations, this paper proposes the second condition to make the algorithm adaptive to different datasets and situations. According to the analysis of the Gap values of non-important points and important points in an interval formed by two adjacent important points, parameter *k* can be manipulated to make a trade-off between the accuracy of the detection algorithm and the number of iterations. We can also use another parameter as the termination conditions, as long as we use a heuristic dynamically adjusted method.

In this method, the distance between points must be obtained. Three distance measurement methods were adopted: Euclidean distance method (ED), Perpendicular distance method (PD), and Vertical distance method (VD).

- The ED measurement method calculates the sum of the Euclidean distance from *P*_*3*_(*x*_*3*_, *y*_*3*_) to *P*_*1*_(*x*_*1*_, *y*_*1*_) and *P*_*2*_(*x*_2,_ *y*_*2*_). The formula is as follows,
  $$ED(p_{3},p_{1},p_{2})=\sqrt{{\left(x_{2}-x_{3}\right)}^{2}+{\left(y_{2}-y_{3}\right)}^{2}}+\sqrt{{\left(x_{1}-x_{3}\right)}^{2}+{\left(y_{1}-y_{3}\right)}^{2}}$$ (5)
  where *P*_*1*,_ *P*_*2*_ and *P*_*3*_ are three two-dimensional points, and *(x*_*i*_, *y*_*i*_ *)* refers to the coordinate of *P*_*i*_ (*i* = 1, 2, 3).
- The PD measurement method calculates the perpendicular distance from *P*_*3*_(*x*_*3*_, *y*_*3*_) to the line between *P*_*1*_(*x*_*1*_, *y*_*1*_) and P_*2*_(*x*_*2*_, *y*_*2*_). *P*_*c*_(*x*_*c*_, *y*_*c*_) is the point that *P*_*3*_ intersects point with a perpendicular line. When *y*_*1*_ *≠y*_*2*_ we can infer *x*_*c*_ *≠x*_3._The formula is as follows,
  $$\begin{array}{l} Slope(p_{1},p_{2})=s=\frac{y_{2}-y_{1}}{x_{2}-x_{1}}\\ \begin{array}{ll} x_{c} & =\frac{x_{3}+(sy_{3})+(sy_{2})-(s^{2}x_{2})}{1+s^{2}}\\ y_{c} & =(sx_{c})-(sx_{2})+y_{2} \end{array}\\ PD(p_{3},p_{c})=\sqrt{{\left(x_{c}-x_{3}\right)}^{2}+{\left(y_{c}-y_{3}\right)}^{2}} \end{array}$$ (6)
  where *s* refers to the slope of the line location *P*_*1*_ and *P*_2._
- The VD measurement method calculates the distance from *P*_*3*_(*x*_*3*_, *y*_*3*_) to the line between *P*_*1*_(*x*_*1*_, *y*_*1*_) and *P*_*2*_(*x*_*2*_, *y*_*2*_) using a line parallel to the y-axis that intersects at *P*_*c*_(*x*_*c*_, *y*_*c*_). The formula is as follows,
  $$VD(p_{3},p_{c})=\left|y_{c}-y_{3}\right|=\left|(y_{1}+(y_{2}-y_{1})\frac{x_{c}-x_{1}}{x_{2}-x_{1}})-y_{3}\right|$$ (7)
  where *P*_*1*_, *P*_*2*_, *P*_*3*_ and *P*_*c*_ are the three two-dimensional points, *(x*_*1*_, *y*_*1*_ *)* is the coordinate of *P*_*1*_, *(x*_*2*,_ *y*_*2*_ *)* is the coordinate of *P*_*2*_, *(x*_*3*_, *y*_*3*_ *)* is the coordinate of *P*_*3*_, and *(x*_*c*_, *y*_*c*_ *)* is the coordinate of *P*_*c*_.

**Fig 3:**
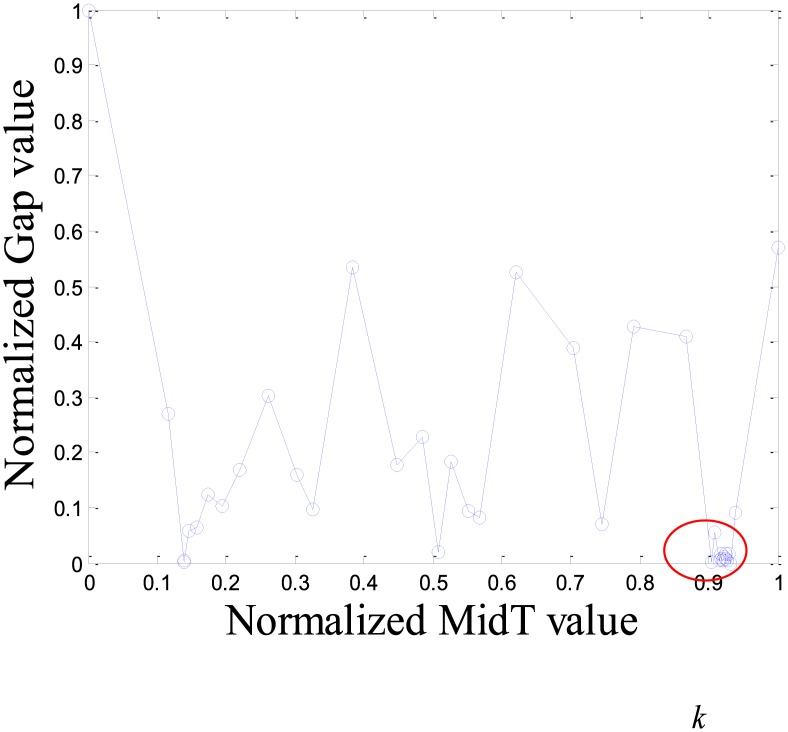
Rating time gaps series of an item in MovieLens.

**Fig 4:**
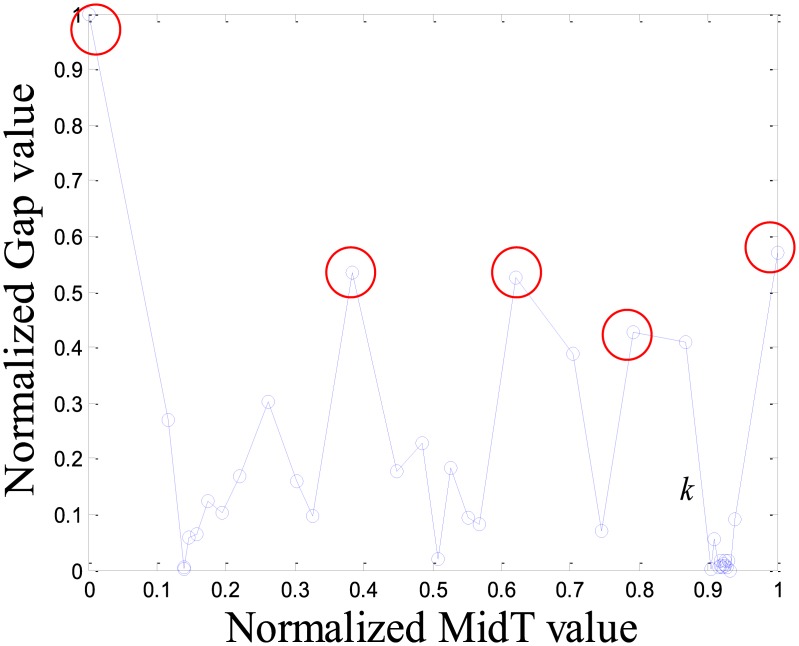
All important points that meet the threshold.

As an example, we select an item randomly from MovieLens. The item’s IRTGS is shown in Fig 3. The points in the red circle are very dense that indicates the item has been rated many times in a short time and it is significant difference with the rest of time intervals. Thus, we suppose there is an abnormal event making the sudden change of the rating number, so we think the points in the red circle most likely are spam ratings. This IRTGS was dynamically partitioned; it determined that the points in the red circles in Fig 4 are the important points. These important points can be directly mapped back to the points in the original IRTS, and then IRTS uses these to divide them into time intervals.

### 3.3 Shilling attack detection method

For a rating-based recommender system, the ratings are discrete values, rather than consecutive values. Therefore, it requires special handling. First, we gather the statistics of the rating values within the time interval. Next, we use the rating relative class distribution to represent the features of the time interval. All time intervals have the same number of features. Therefore, a similarity measurement will be taken for comparison and to establish a threshold to identify suspicious intervals. In this paper, chi square distribution (χ^2^) [18] is utilized to measure the similarity of the rating class distribution in time intervals and then to identify the time intervals that exceed a threshold. χ^2^ compares two or more sample rates and is able to obtain the rating relative classes’ distribution similarity between the two time intervals via analyzing the correlation of two classified variables. χ^2^ is a nonparametric test, and it decreases the reliance of algorithms upon prior inputs; it just requires setting value α according to significance levels. The equation for χ^2^ is shown as Formula (8),

$$\chi^{2}=\sum_{i=1}^{m}\sum_{j=1}^{r}\frac{{\left(A_{ij}-E_{ij}\right)}^{2}}{E_{ij}}$$ (8)

where *m* refers to the comparison intervals number (*m* equals 2), *r* refers to the quantity of relative classes, *A*_*ij*_ refers to the quantity of the *j*-th relative class in the *i*-th time interval, *E*_*ij*_ equals *R*_*i*_ *× C*_*j*_ */N*, *R*_*i*_ refers to the sum of all relative classes in the *i*-th time interval, *C*_*j*_ refers to the total number of the *j*-th relative class in the two intervals, and *N* refers to the sum of all *C*_*j*_.

According to the significance levels’ α, we obtain the related boundary value, namely a threshold. Comparing χ^2^ of each time interval with the threshold, we obtain the abnormal intervals that exceed the threshold. The significance levels and related boundary values are shown in Table 2 [18].

**Table 2:** Significance levels and related boundary values.

**Significance levels**	0.25	0.10	0.05	0.025	0.01	0.005
**boundary values**	5.385	7.779	9.488	11.143	13.277	14.860

## Experiments

### 4.1 Experimental dataset

In the experiments, we use the commonly utilized MovieLens dataset [17, 18, 25, and 26]. MovieLens has been collected by GroupLens Research and made available from the GroupLens web site (http://grouplens.org/datasets/movielens/). MovieLens is a recommender system and a virtual community website; its main function is recommending movies according to users’ preferences. This function is accomplished utilizing a collaborative filtering technique. When a new user enters MovieLens, they need to rate 15 movies, and the score range is from 1 to 5. Along with detailed time information, labeling is another important application in MovieLens. Users can add tags to a film, obtain movie information, and search for a film according to tags added by other users. MovieLens consists of three different datasets sizes.

MovieLens 100K: This is the minimal MovieLens data set, including 100,000 ratings (1–5 marks) from 943 users on 1,682 movies. Each user has rated at least 20 movies.
MovieLens 1M: This is a medium-size MovieLens dataset. The dataset consists of 1,000,000 ratings (1–5 marks) from 6,040 users on 3,900 movies.
MovieLens 10M: This is the largest MovieLens dataset, including 100,000 tags and 10,000,000 ratings (1–5 marks) from 71,567 users on 10,681 movies.

All of the datasets provide rating time and attributes of users and items.

### 4.2 Experimental procedures

In this research experiment, we used two datasets: 1) the sparse MovieLens 100k dataset named Dataset-100K; and 2) a dense dataset, named Dataset-1M, including all of the items with at least 500 ratings (618 items in total) from the MovieLens 1M dataset.

**Fig 5:**
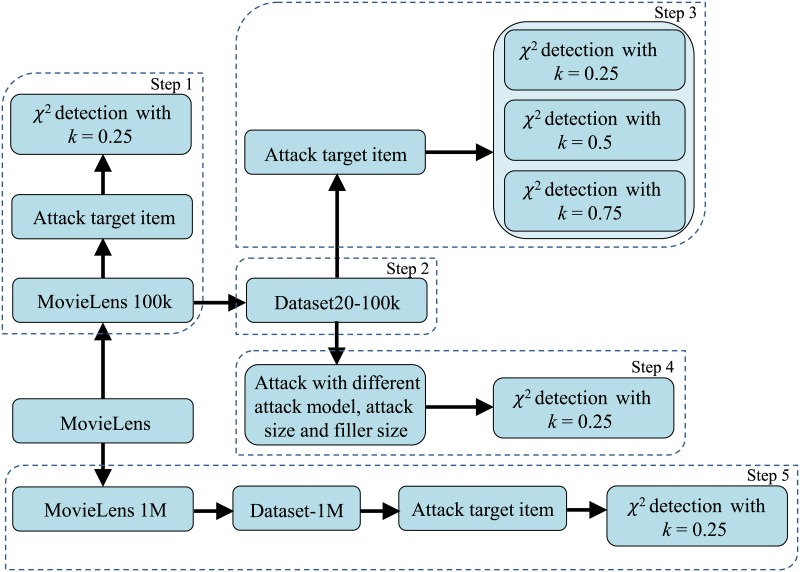
Steps of the experiments.

The experimental steps are shown as Fig 5.

Step 1: Conduct the detection experiments on the MovieLens 100k with k = 0.25. In the experiment, we only attack the target item.
Step 2: Obtain Dataset20-100k according to the MovieLens 100k.
Step 3: Conduct the experiments on the Dataset20-100K with k = 0.25, k = 0.5 and k = 0.75, respectively. In this part, we only attack the target item.
Step 4: Conduct an experiment on the Dataset20-100K with different attack models, attack sizes, and filler sizes. In the experiment k = 0.25.
Step 5: To facilitate comparison with others’ research, we select part of the data from MovieLens 1M to compose a new dataset named Dataset-1M. Here we only attack the target items. The detection experiments on the Dataset-1M use k = 0.25.

### 4.3 Select *k* value of the dynamic partition for time series

The distance measurement was a critical step to find important points in the time series. To verify the validity of ED, PD and VD (mentioned in section 3.2) toward the time series dynamic partition, this paper conducts experiments on MovieLens 100K and MovieLens 1M. In these experiments, we change the *k* value to calculate ADNI. The purpose is not only to test whether the distance calculation method would group those unimportant points together but also to keep a significant distance off the two important points of the time interval.

At the beginning, we conduct the experiments on all items. Then, we divide all items into four types: fad items, fashion items, style items and scallop items [18].

**Fig 6:**
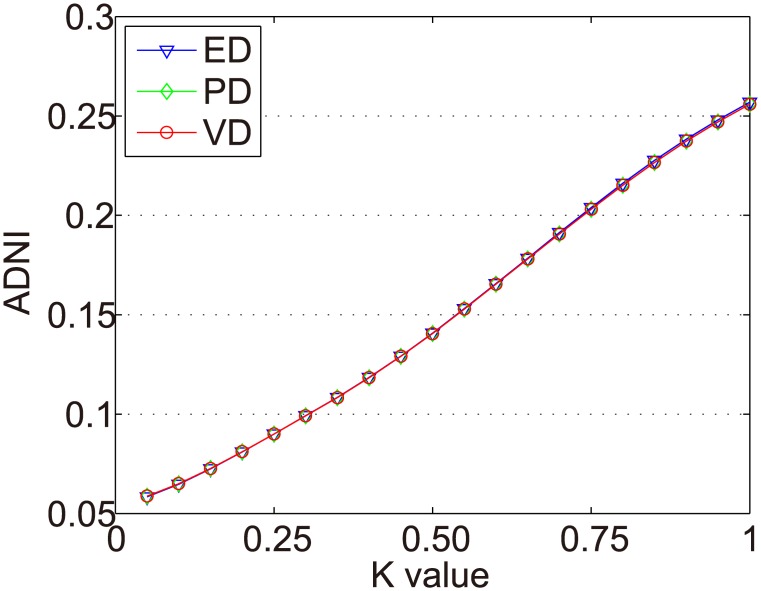
ADNIs of all items in Dataset-100k.

The ADNIs of all items in Dataset-100k are shown as Fig 6. The x axis represents the *k* value (0 < *k* < 1), and the y axis represents ADNI.

**Fig 7:**
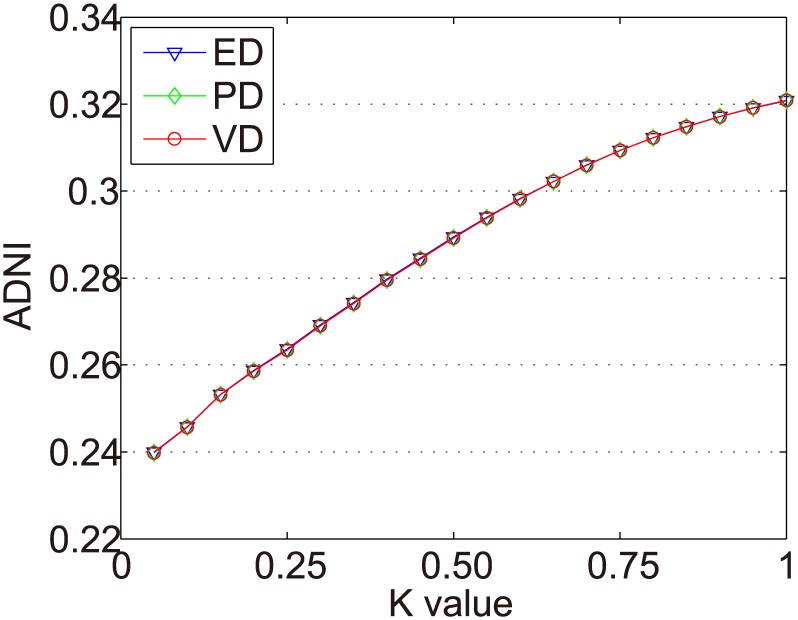
ADNIs of the fad items in Dataset-100k.

**Fig 8:**
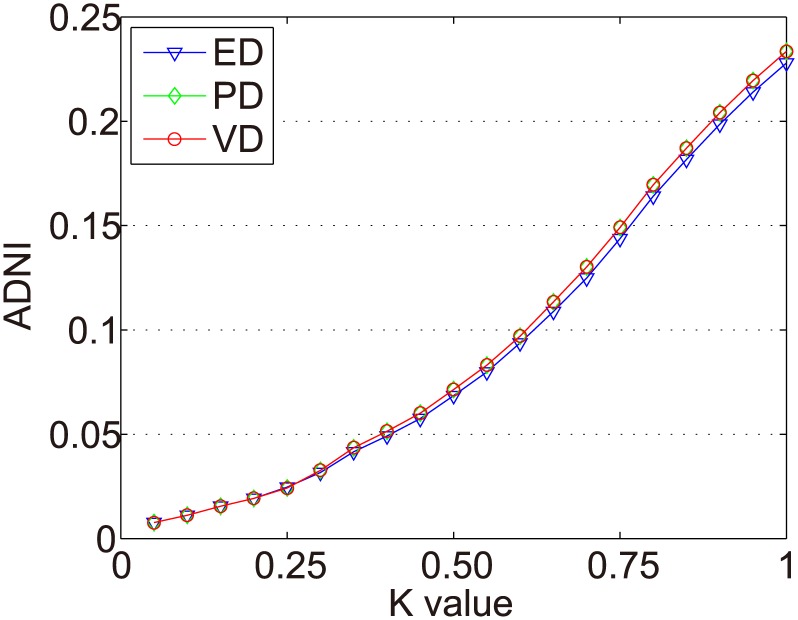
ADNIs of the fashion items in Dataset-100k.

**Fig 9:**
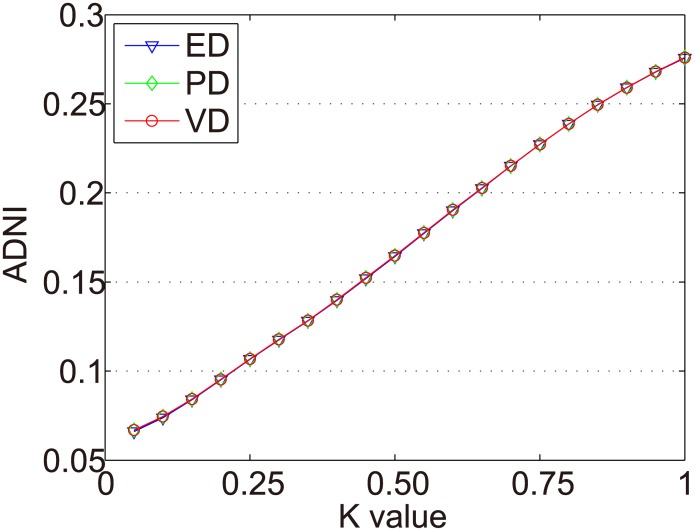
ADNIs of the style items in Dataset-100k.

**Fig 10:**
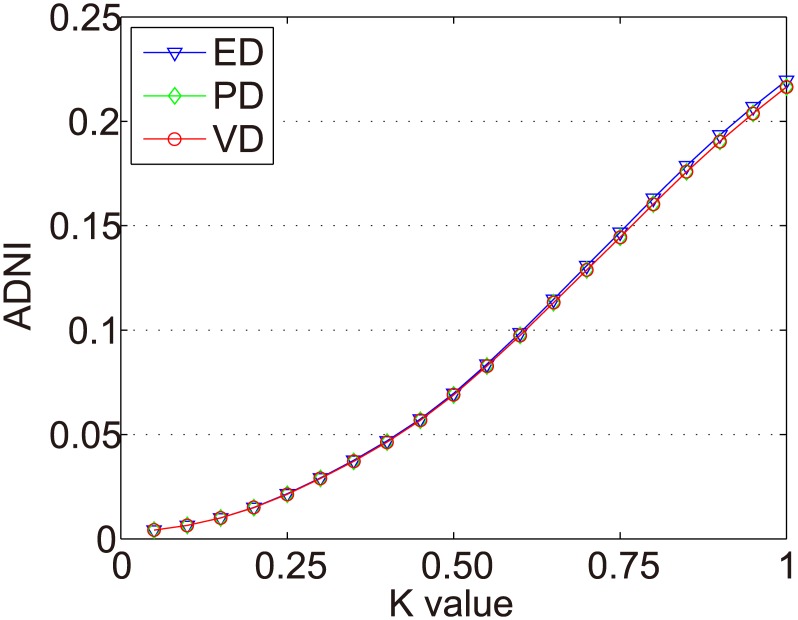
ADNIs of the scallop items in Dataset-100k.

In Figs 7–10 we show the ADNIs for the four item types (fad, fashion, style, and scallop) in Dataset-100K. The x and y axes represent the same values as in Fig 6.

**Fig 11:**
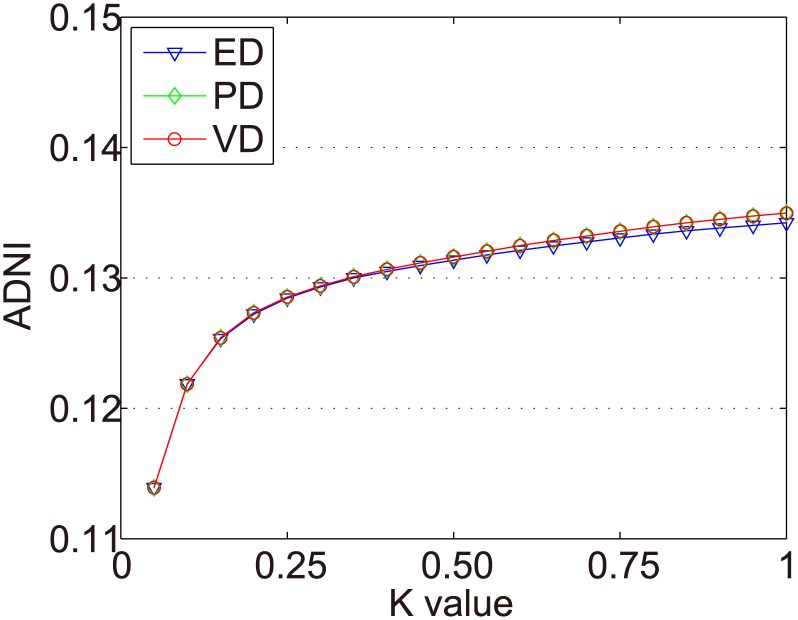
ADNIs of all items in Dataset-1M.

**Fig 12:**
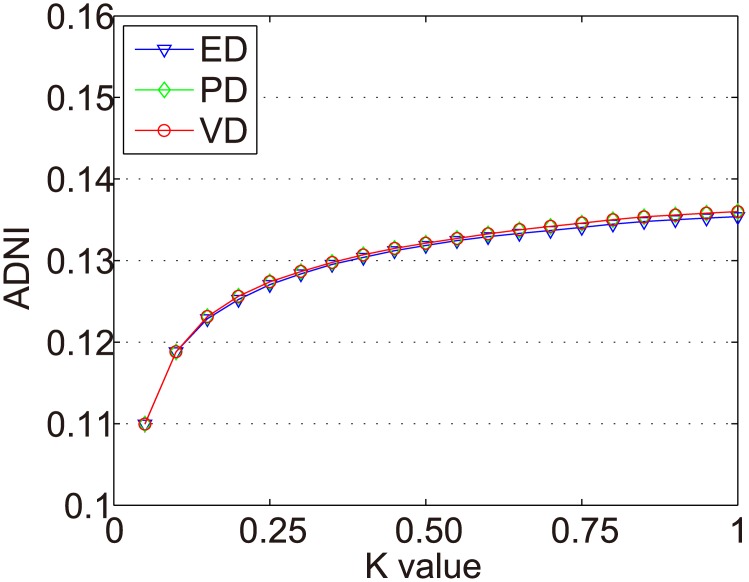
ADNIs of the fashion items in Dataset-1M.

**Fig 13:**
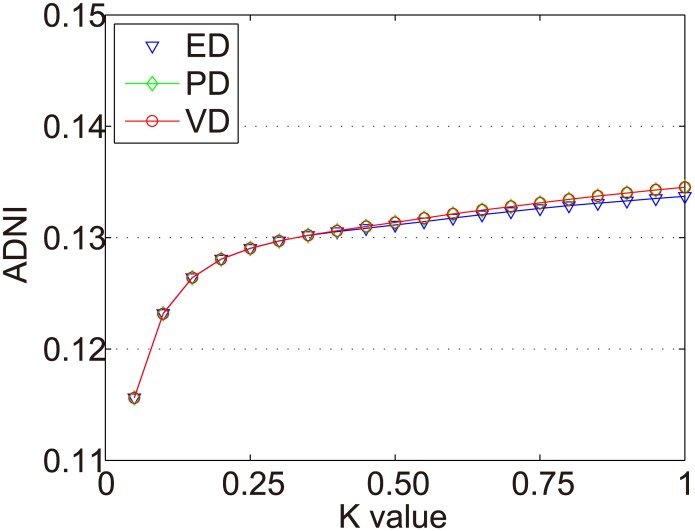
ADNIs of the scallop items in Dataset-1M.

In the Dataset-1M, the same items were selected in the experiments of Zhang et al. [17]. We use all items with at least 500 ratings (618 items in total) to conduct the experiments. There are 187 fashion items and 431 scallop items. The ADNIs of all items in the Dataset-1M are shown as Fig 11. The ADNIs of fashion items and scallop items in Dataset-1M are shown as Figs 12 and 13. The x and y axes represent the same values as in Fig 6.

The purpose of dynamic partition is to divide close rating times into one time interval and to provide data for the next irregular time interval detection. However, the larger the *k* value, the fewer the time intervals. Thus, the window size for each time interval is larger and the difference of points in one time intervals greater. This leads to an increase in the ADNI of each time interval. The smaller the *k* value, there are more time intervals and it will lead to the detection time increase. The window size for each time interval is smaller and the difference of the points in one time intervals is less. Thus, the ADNI of each time interval will decrease. Therefore, the optimal *k* value should be set when the average distance changes from fast to slow. The optimal *k* value meets two requirements: 1) generates the needed number of time intervals, and 2) is able to combine close rating times into one time interval. Figs 6–10 show that in a sparse dataset (such as Dataset-100k), the ADNI will not turn stable as *k* changes. Thus, *k* is set to 0.25, 0.5, and 0.75, respectively, in the detection experiments on Dataset-100k. Figs 11–13 show that in a dense dataset (such as Dataset-1M), when *k* equals 0.25, the three ADNI curves of ED, PD, and VD will turn smooth. Consequently, if the dataset is dense, 0.25 is the optimal value for conducting these experiments.

### 4.4 Abnormal items detection method based on time series dynamic partition

Because the ratings are sparse in real recommendation systems, without loss of generality, we conduct detection experiments on the Dataset-100k. First, we simulate attacks under different attack models, attack sizes, and filler sizes on random selected items as well as dynamically partitioned time series for each item. Second, we use the metrics of the detection rate and the false alarm rate [17–18] to evaluate the detection results. The detection rate (9) is defined as the number of detected attacking events divided by the number of the total attacking events. Here, a detected attacking event means that an abnormal time interval is detected. The false alarms rate (10) refers to the number of normal intervals that are recognized as abnormal time intervals, which we can calculate by dividing the number of false alarms by all normal intervals.

$$DetectionRate=\frac{NumberOfDetectedAttackEvents}{NumberOfTotalAttackEvents}$$ (9)

$$FalseAlarmRate=\frac{NumberOfFalseAlarms}{NumberOfNormalIntervals}$$ (10)

According to the features of shilling attack models, which are mentioned in Section 2.2, we assume that the attack profiles are inserted in a relatively short period [17]. Additionally, the detection method in this paper only considers the ratings on items since the change of attack model and filler size have little effect on detection results. To validate this characteristic of this method, we carry out a series of experiments under different attack models and filler size. The filler size will equal one item or change from 1% to 10%. The attack size will change from 5 to 50 (which is approximately 0.5% to 5%), 1% to 10%, and 1% to 15%.

#### 4.4.1 Detection on Dataset-100k

To show the detection results, we first show the experiments under attack with one-item filler size. In these experiments, we inject ratings using five (for push attacks) or one (for nuke attacks); the *k* value is set to 0.25. We randomly select twenty target items. The attack sizes for each target item (attack size refers to the number of attacks) varies from 5 to 50. We repeat these experiments twenty times for each attack size. We set the average detection rate and false alarm rate of these experiments to the final detection rate and false alarm rate.

**Fig 14:**
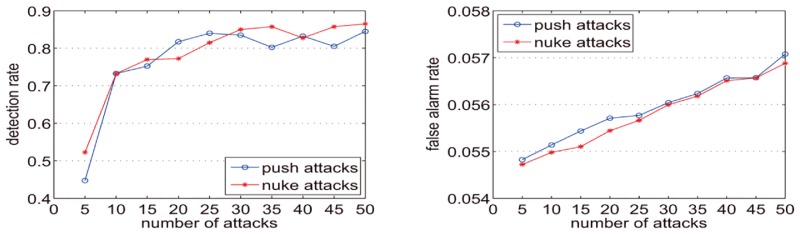
Detection rates and false alarm rates for DataSet-100k when k = 0.25.

In Fig 14, the y-axis represents the detection rate and the false alarm rate. The x-axis represents the number of attacks, which is also the number of injected spam users. Fig 14 shows that when the number of attack profiles is 5 (attack size is approximately 0.05%) the detection rate is less than 0.45 for push attack and 0.55 for nuke attack. However, when the number of attack profiles is 10 (attack size is approximately 1%) the detection rate for both push attack and nuke attack exceeds 70%, and the detection rate exceeds 80% when the number of attack profiles is 25 (attack size is approximately 3%). With an increase in the number of attack profiles, the detection rate slightly fluctuates, but the overall trend is increasing. With the expansion of the attack size, the false alarm rate creeps up modestly. In the detection experiments on nuke attacks, the false alarm rates increases slightly, from 0.0547 to 0.0569. Additionally, in the push attack detection experiments the false alarm rate increases slightly from 0.0548 to 0.0571.

#### 4.4.2 Detection on Dataset-100k with at least 20 rating

Dataset-100k has 44% of items with fewer than 20 ratings. To further evaluate the capability of the method we select the items with at least 20 ratings from MovieLens 100K (named Dataset20-100k) for the following experiments. We conduct the dynamic partitioning separately for item-rating series and corresponding shilling attack detection experiments when *k* = 0.25, *k* = 0.5, and *k* = 0.75. In the experiments, we randomly select twenty target items. For each target item, the rating was 5 (for push attacks) or 1 (for nuke attacks), and the attack sizes vary from 1% to 10%. For each attack size, we repeat the experiments twenty times. The final detection rate and false alarm rate are set according to the average values of the experiments.

**Fig 15:**
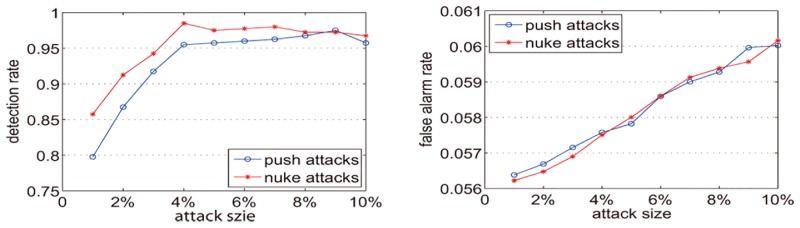
Detection rates and false alarm rates for Dataset20-100k with k = 0.25.

**Fig 16:**
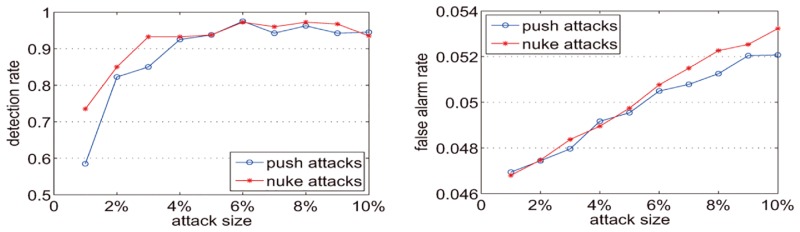
Detection rates and false alarm rates for Dataset20-100k with k = 0.5.

**Fig 17:**
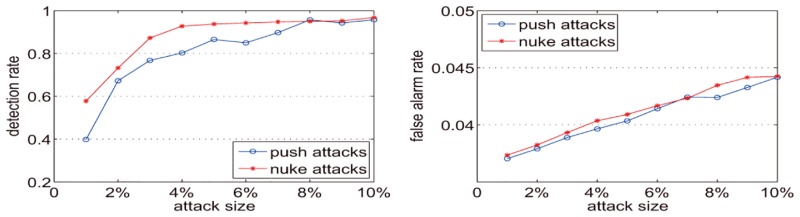
Detection rates and false alarm rates for Dataset20-100k with k = 0.75.

Fig 15 shows that when *k* is 0.25 and the attack size is 1%, the detection rate is nearly 80% for push attacks and is greater than 85% for nuke attacks. When the attack size is not less than 3%, the detection rates on push attacks and nuke attacks are both greater than 90%. Although the attack size exceeds 4%, the detection rates on push attacks and nuke attacks are greater than 95%. With the attack size increase, the false alarm rate increases slightly. For push attacks, the false alarm rates increase from 0.0564 to 0.06. For nuke attacks, the false alarm rates increase from 0.0562 to 0.0602. Fig 16 shows that when k is 0.5 and the attack size is 1%, the detection rate for the push attack is less than 60% and for the nuke attack is less than 75%. When the attack size is 3% the detection rate for the push attack is less than 90% and for the nuke attack it is more than 90%. When the attack size is not less than 4% the detection rates for both the push attack and the nuke attack are more than 90%. However in most cases, the detection rates in Fig 16 are less than those in Fig 15. Like Fig 15, the false alarm rate in Fig 16 increases slightly with the increase in attack size. For the push attack, the false alarm rates increase from 0.0469 to 0.0521. For the nuke attack, the false alarm rates increase from 0.0468 to 0.0532. Fig 17 shows that when k is 0.75 and attack size is 1%, the detection for the push attack is less than 40% and for the nuke attack is less than 60%. When the attack size is not less than 4%, the detection rates for the nuke attack are more than 90%, but for the push attack the detection rates are more than 90% when the attack size is not less than 8%. However, in most cases, the detection rates in Fig 17 are less than Figs 15 and 16. For the push attack, the false alarm rates increase from 0.037 to 0.0442. For the nuke attack, the false alarm rates increase from 0.0373 to 0.0442. Figs 15–17 illustrate that with the increase of k, the detection rate and false alarm rate are both reduced. Since with the *k* increase the number of time intervals is decreased, the number of detected abnormal time intervals is reduced, and the detection rate and false alarm rate are correspondingly decreased.

#### 4.4.3 Detection for shilling attacks for different attack models

To evaluate the results of the proposed method, changing the attack model or filler size has little effect on detection results. We simulate four types of attack models on the items. The attack sizes were, respectively, 1%, 3%, 7%, 10% and 15%, and the filler sizes were, respectively, 1%,3%,7% and 10%. We conduct push attack and nuke attack experiments.

Table 3 presents the detection rate and false alarm rate results for the four types of attack models. Where Rand attacks represent random attack, Avg attack is average attack, Band attack refers to bandwagon attack, and Seg attack refers to segment attack. Table 3 shows that detection rates increase when attack size increases and slightly fluctuates with the filler size increase. The detection rates of nuke attacks are generally higher than those of push attacks. When the attack size is 1% the detection rates for the push attack is more than 70% and for the nuke attack they are more than 80% for the four attack types. When attack size is not less than 3%, the detection rates for both nuke and push attacks are more than 90% for the four kinds of attack. In addition, the best detection rate is nearly 99% for the four kinds of attacks. For random attack types, the false alarm rates for the push attack increases from 0.0563 to 0.0628 and for the nuke attack increases from 0.0562 to 0.0624. Looking at the average attack type, the false alarm rates for the push attack increases from 0.0565 to 0.0639 and for the nuke attack increases from 0.0563 to 0.0636. Now for the bandwagon attack type, the false alarm rates for the push attack increase from 0.0564 to 0.0639 and for nuke attack increase from 0.0563 to 0.0636. We see for the segment attack type that the false alarm rates for push attack increase from 0.0571 to 0.0907 and for nuke attack increase from 0.057 to 0.0913. The false alarm rates of the segment attack are generally a little higher than those of the other attack models, but the detection rates for the four attack models are slightly different. Additionally, the experimental results show that the detection rates and false alarm rates changed little with the changing of the filler size. Thus, the experimental results indicate that with this approach, changing the attack model or filler size has little effect on detection results.

**Table 3:** Detection rates and false alarm rates for four typical shilling attacks.

Attack type	Attack size	Filler size	Detection rate				False alarm rate			
			Rand attack	Avg attack	Band attack	Seg attack	Rand attack	Avg attack	Band attack	Seg attack
**Push Attack**	1%	1%	0.7346	0.7386	0.7341	0.7705	0.0564	0.0565	0.0564	0.0571
		3%	0.7727	0.7614	0.7886	0.7341	0.0563	0.0566	0.0565	0.0580
		7%	0.7591	0.7545	0.7591	0.7000	0.0565	0.0571	0.0565	0.0599
		10%	0.7591	0.7682	0.8023	0.7636	0.0566	0.0576	0.0564	0.0608
	3%	1%	0.9545	0.9318	0.9432	0.9364	0.0571	0.0575	0.0574	0.0587
		3%	0.9259	0.9500	0.9386	0.9477	0.0574	0.0583	0.0575	0.0615
		7%	0.9432	0.9409	0.9318	0.9545	0.0573	0.0586	0.0576	0.0653
		10%	0.9409	0.9295	0.9364	0.9386	0.0575	0.0581	0.0574	0.0678
	7%	1%	0.9773	0.9795	0.9591	0.9727	0.0591	0.0601	0.0598	0.0621
		3%	0.9568	0.9705	0.9659	0.9591	0.0591	0.0607	0.0596	0.0673
		7%	0.9705	0.9636	0.9636	0.9818	0.0589	0.0592	0.0585	0.0734
		10%	0.9795	0.9659	0.9727	0.9614	0.0578	0.0586	0.0585	0.0764
	10%	1%	0.9727	0.9682	0.9659	0.9773	0.0607	0.0619	0.0617	0.0646
		3%	0.9773	0.9705	0.9841	0.9841	0.0606	0.0617	0.0615	0.0703
		7%	0.9727	0.9682	0.9909	0.9750	0.0591	0.0598	0.0603	0.0776
		10%	0.9841	0.9818	0.9568	0.9727	0.0587	0.0584	0.0598	0.0820
	15%	1%	0.9727	0.9705	0.9659	0.9818	0.0628	0.0635	0.0639	0.0678
		3%	0.9864	0.9864	0.9795	0.9841	0.0621	0.0639	0.0630	0.0766
		7%	0.9750	0.9818	0.9886	0.9841	0.0601	0.0596	0.0622	0.0841
		10%	0.9886	0.9705	0.9841	0.9773	0.0597	0.0573	0.0622	0.0907
	1%	1%	0.8457	0.8614	0.8423	0.8636	0.0564	0.0563	0.0564	0.0570
		3%	0.8582	0.8705	0.8326	0.8614	0.0562	0.0566	0.0564	0.0580
		7%	0.8298	0.8523	0.8614	0.8773	0.0566	0.0569	0.0564	0.0596
		10%	0.8376	0.8682	0.8457	0.8795	0.0567	0.0572	0.0563	0.0607
	3%	1%	0.9364	0.9568	0.9318	0.9727	0.0571	0.0573	0.0574	0.0588
		3%	0.9273	0.9750	0.9409	0.9659	0.0574	0.0583	0.0575	0.0615
		7%	0.9545	0.9636	0.9500	0.9500	0.0573	0.0580	0.0578	0.0646
		10%	0.9500	0.9636	0.9273	0.9750	0.0575	0.0588	0.0575	0.0680
	7%	1%	0.9727	0.9636	0.9682	0.9886	0.0589	0.0598	0.0598	0.0621
		3%	0.9523	0.9818	0.9659	0.9727	0.0594	0.0603	0.0596	0.0672
		7%	0.9545	0.9705	0.9795	0.9773	0.0586	0.0596	0.0594	0.0730
		10%	0.9682	0.9682	0.9727	0.9841	0.0583	0.0583	0.0584	0.0761
	10%	1%	0.9727	0.9841	0.9727	0.9864	0.0611	0.0617	0.0616	0.0645
		3%	0.9727	0.9818	0.9750	0.9705	0.0607	0.0627	0.0616	0.0711
		7%	0.9795	0.9841	0.9727	0.9886	0.0592	0.0600	0.0605	0.0775
		10%	0.9682	0.9750	0.9773	0.9750	0.0584	0.0581	0.0598	0.0819
	15%	1%	0.9659	0.9727	0.9727	0.9795	0.0624	0.0633	0.0636	0.0687
		3%	0.9727	0.9841	0.9864	0.9682	0.0622	0.0636	0.0632	0.0759
		7%	0.9818	0.9773	0.9795	0.9773	0.0600	0.0613	0.0618	0.0842
		10%	0.9841	0.9727	0.9795	0.9841	0.0595	0.0589	0.0620	0.0913

#### 4.4.4 Analysis of experimental results

The results in section 4.4.2 demonstrate that the detection rate is greater than 90% when *k* is 0.25 and attack size is greater than 3%. The false alarm rates fluctuate within a range of 0.01, and the highest false alarm rate is less than 0.0666. Therefore, the proposed method has higher detection rates and lower false alarm rates.

The experimental results in section 4.4.3 indicate that changing the attack model or filler size has little effect on detection results. The experimental results show that the proposed method has high detection rates, low false alarm rates, and high stability. A possible reason for high detection rates and low false alarm rates is the dynamic partition on time series for item rating. A possible reason for the high stability is that in the detection of abnormal time intervals of an item, only the ratings on the item are used; therefore, the changing of the attack model or filler size has little effect on detection results.

### 4.5 Comparison of related work

Many detection approaches [25–27] achieved good results in detecting spam users; however, the purpose of our proposed method is different from these approaches. This method is used for the detection of abnormal items. Therefore, these works will not be compared. Only two methods [17–18] were compared for abnormal items detecting.

**Fig 18:**
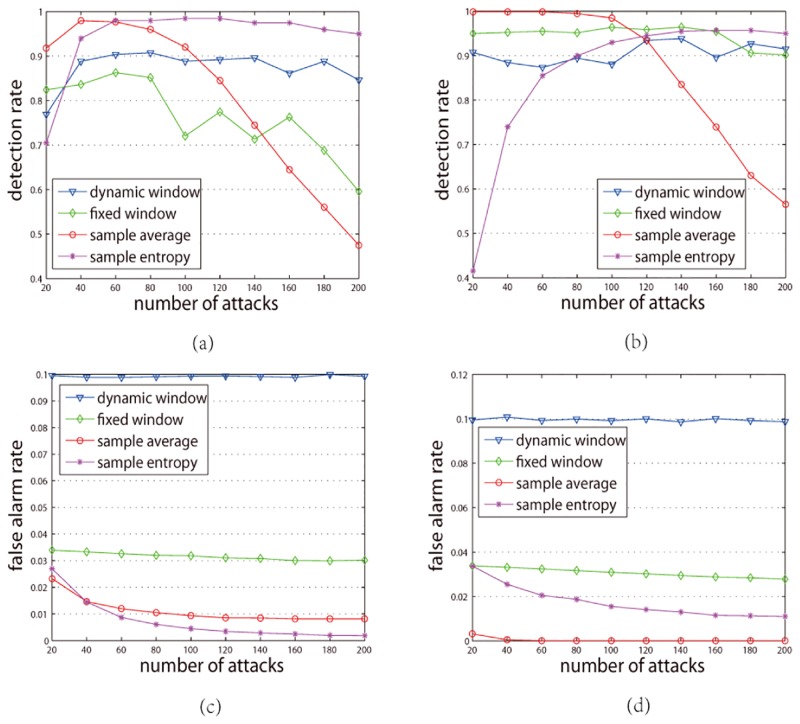
Detection rates and false alarm rates on the Dataset-1M dataset ((a), (b), (c) and (d)). Dynamic window refers to the detection results of our method, fixed windows represents the detection results of Gao's method, sample average and sample entropy refer to the detection results of Zhang's method, (a) detection rate for push attacks, (b) detection rate for nuke attacks, (c) false alarm rate for push attacks, (d) false alarm rate for nuke attacks.

To compare with the experimental results of Zhang et al. [17] and Gao et al. [18], we conduct the experiment using the Dataset-1M. The experimental results are shown as Fig 18.

For push attacks: Fig 18a shows that the detection rates of our method (dynamic window) are relatively stable when the number of attacks increases and when number of attacks more than 200 the detection rate is greater than 0.8. The detection rates of Gao (fixed window) fluctuant when the number of attacks increases and the maximum less than 0.9. When the number of attacks is greater than 200, the detection rate is less than 0.6. When the number of attacks is 20, the detection rate of sample entropy (Zhang's method) is 0.7 less than our method's (our method is 0.77), but the detection rates of the sample entropy increase significantly when the number of attacks increase. However, the detection rates of the sample average (Zhang's method) decreases significantly when the number of attacks increases. When the number of attacks is more than 200, the detection rate of the sample average is less than 0.5.

For nuke attacks: Fig 18b shows that the detection rates of our method (dynamic window) are relatively stable when the number of attacks increase and when number of attacks are more than 200 the detection rate is greater than 0.9. In most cases, the detection rates of our method are more than 0.9. When number of attacks is 20, the detection rate of sample entropy is 0.42 and when number of attacks is less than 80, the detection rates of sample entropy are less than our method's. When number of attacks is more than 120, the detection rates of sample average are less than our method's. The detection rate of sample entropy is less than o.6 when the number of attacks is 200. In most cases, the detection rates of the fixed window are more than our method's. However, when the number of attacks is more than 180, our method's detection rates are more than Gao's.

Fig 18c and 18d show that the false alarm rates of the three methods are small. On the other hand, the false alarm rates of our method are slightly higher than the other two methods. For push attacks: the false alarm rates of our method are about 0.099 while those of Zhang's method are less than 0.03 and those of Gao's method are less than 0.04. For nuke attacks: the false alarm rates of our method are about 0.1 while those of Zhang's method are less than 0.04 and those of Gao's method are less than 0.04.

## Conclusions and Future Work

This paper proposes a dynamic partition approach for item-rating time series based on important points. The approach first finds the important points in IRTGS to partition the time intervals. It then calculates χ^2^ for each time interval and marks the time intervals abnormal if χ^2^ exceeds a threshold. When detecting an item, only the ratings of the item will be used in the proposed approach; therefore, the changing of the attack model or filler size has little effect on the detection results. To evaluate the proposed approach, experiments were conducted using different attack models, attack sizes, and filler sizes. The experimental results show that the approach has a high detection rate, a low false alarm rate, and high stability.

Compared to other methods, the false alarm rate of the detection method this paper proposes is slightly higher. The false alarm rates of this method are higher than those of others that may because the approach does not find an ideal value for significance level and relative threshold, and that will lead to more normal time intervals are misjudged as abnormal intervals. In the future work, we will try to reduce the false alarm rate. Besides the detection approach was focused on abnormal items. Next, the proposed method will be incorporated into classical shilling attack algorithms to detect spam users because both the detection approach of spam users and of abnormal items are the two most important aspects of researching shilling attacks.

## References

[1] Mehta B, Nejdl W. Unsupervised strategies for shilling detection and robust collaborative filtering. User Modeling and User-Adapted Interaction, 2009, 19(1–2): 65–97.

[2] Zhang ZK, Liu C, Zhang YC, Zhou T. Solving the cold-start problem in recommender systems with social tags J. EPL (Europhysics Letters), 2010, 92(2): 28002.

[3] Zhou T, Kuscsik Z, Liu JG, Medo M, Wakeling JR, Zhang YC. Solving the apparent diversity-accuracy dilemma of recommender systems J. Proceedings of the National Academy of Sciences, 2010, 107(10): 4511–4515. 10.1073/pnas.1000488107 PMC2842039 20176968

[4] Palmisano C, Tuzhilin A, Gorgoglione M. Using context to improve predictive modeling of customers in personalization applications. Knowledge and Data Engineering, IEEE Transactions on, 2008, 20(11): 1535–1549.

[5] Zhou T, Medo M, Cimini G, Zhang ZK, Zhang YC. Emergence of scale-free leadership structure in social recommender systems J. PLoS One, 2011, 6(7): e20648 doi: 10.1371/journal.pone.0020648 21857891 10.1371/journal.pone.0020648 PMC3152579

[6] Rong W, Peng B, Ouyang Y, Liu K, Xiong Z. Collaborative personal profiling for web service ranking and recommendation J. Information Systems Frontiers, 2014: 1–18.

[7] Dellarocas C. Immunizing online reputation reporting systems against unfair ratings and discriminatory behavior. In: Proceedings of the 2nd ACM conference on Electronic commerce. ACM, 2000: 150–157.

[8] O'Mahony M, Hurley N, Kushmerick N, Silvestre G. Collaborative recommendation: A robustness analysis. ACM Transactions on Internet Technology (TOIT), 2004, 4(4): 344–377.

[9] Mehta B, Hofmann T, Fankhauser P. Lies and propaganda: detecting spam users in collaborative filtering. In: Proceedings of the 12th international conference on Intelligent user interfaces. ACM, 2007: 14–21.

[10] Chirita PA, Nejdl W, Zamfir C. Preventing shilling attacks in online recommender systems In: Proceedings of the 7th annual ACM international workshop on Web information and data management. ACM, 2005: 67–74.

[11] Burke R, Mobasher B, Williams C, Bhaumik R. Classification features for attack detection in collaborative recommender systems. In: Proceedings of the 12th ACM SIGKDD international conference on Knowledge discovery and data mining. ACM, 2006: 542–547.

[12] Williams C, Mobasher B. Profile injection attack detection for securing collaborative recommender systems. DePaul University CTI Technical Report, 2006: 1–47.

[13] Hurley N, Cheng Z, Zhang M. Statistical attack detection. In:Proceedings of the third ACM conference on Recommender systems. ACM, 2009: 149–156.

[14] Mehta B. Unsupervised shilling detection for collaborative filtering. In: Proceedings of the national conference on Artificial Intelligent. Menlo Park, CA; Cambridge, MA; London; AAAI Press; MIT Press; 1999, 2007, 22(2): 1402.

[15] Burke R, Mobasher B, Bhaumik R, Williams C. Segment-based injection attacks against collaborative filtering recommender systems. In: Data Mining, Fifth IEEE International Conference on. IEEE, 2005: 4 pp.

[16] Bryan K, O'Mahony M, Cunningham P. Unsupervised retrieval of attack profiles in collaborative recommender systems. In: Proceedings of the 2008 ACM conference on Recommender systems. ACM, 2008: 155–162.

[17] Zhang S, Chakrabarti A, Ford J, Makedon F. Attack detection in time series for recommender systems. In: Proceedings of the 12th ACM SIGKDD international conference on Knowledge discovery and data mining. ACM, 2006: 809–814.

[18] Gao M, Yuan Q, Ling B, Xiong Q. Detection of Abnormal Item Based on Time Intervals for Recommender Systems. The Scientific World Journal, 2014, 2014. 10.1155/2014/845897 PMC3945428 24693248

[19] Bobadilla J, Ortega F, Hernando A, Gutiérrez A. Recommender systems survey. Knowledge-Based Systems, 2013, 46: 109–132.

[20] Mobasher B, Burke R, Williams C, Bhaumik R. Analysis and detection of segment-focused attacks against collaborative recommendation In: Nasraoui O., Zaïane O., Spiliopoulou M., Mobasher B., Masand B. & Yu P. (Eds.), Advances in Web Mining and Web Usage Analysis. Springer Berlin Heidelberg, 2006: 96–118.

[21] Burke R, Mobasher B, Zabicki R, Bhaumik R. Identifying attack models for secure recommendation. In: Beyond Personalization: A Workshop on the Next Generation of Recommender Systems. 2005.

[22] Mobasher B, Burke R, Bhaumik R, Williams C. Effective attack models for shilling item-based collaborative filtering systems. In: Proceedings of the 2005 WebKDD Workshop, held in conjuction with ACM SIGKDD’2005. 2005.

[23] Wu Z, Wu J, Cao J, Tao D. HySAD: a semi-supervised hybrid shilling attack detector for trustworthy product recommendation. In: Proceedings of the 18th ACM SIGKDD international conference on Knowledge discovery and data mining. ACM, 2012: 985–993.

[24] Pavlidis T, Horowitz SL. Segmentation of plane curves. IEEE transactions on Computers, 1974, 23(8): 860–870.

[25] Cao J, Wu Z, Mao B, Zhang Y. Shilling attack detection utilizing semi-supervised learning method for collaborative recommender system. World Wide Web, 2013, 16(5–6): 729–748.

[26] Zhang F, Zhou Q. HHT–SVM: An online method for detecting profile injection attacks in collaborative recommender systems. Knowledge-Based Systems, 2014, 65: 96–105.

[27] Bilge A, Ozdemir Z, Polat H. A Novel Shilling Attack Detection Method. Procedia Computer Science, 2014, 31: 165–174.

